# Longitudinal Analysis of Driving Behavior and Cognitive Performance in Older Adults

**DOI:** 10.1093/geroni/igaf122.3288

**Published:** 2025-12-31

**Authors:** Karla Lynch, Melissa Hatch, Jun Ha Chang, Elizabeth Vlock, Matthew Rizzo

**Affiliations:** University of Nebraska Medical Center, Omaha, Nebraska, United States; University of Nebraska Medical Center, Omaha, Nebraska, United States; University of Nebraska Medical Center, Omaha, Nebraska, United States; University of Nebraska Medical Center, Omaha, Nebraska, United States; University of Nebraska Medical Center, Omaha, Nebraska, United States

## Abstract

The early detection of cognitive impairment is vital to effective intervention and treatment. Real-world driving behavior may serve as an indicator of cognitive function. This study examines longitudinal changes in driving behaviors and cognitive performance. Twenty-two older adults (mean age = 74.15 years; 10 females) were assessed at baseline and at follow-up (range: 4.75–7.24 years; mean = 5.41, SD = 0.67). Cognitive function was evaluated using the Montreal Cognitive Assessment (MoCA). Driving behavior was monitored via vehicle GPS sensors over a three-month period at both time points. Maximum distance from home was defined as the vehicle’s displacement at the end of each drive, regardless of where the drive began. Approximately 97,539 miles were recorded. At baseline, the maximum distance from home averaged 466.27 miles (SD = 231.21), which declined to 160.69 miles (SD = 270.27) at follow-up. MoCA scores decreased from a mean of 26.73 (SD = 2.23) at baseline to 25.59 (SD = 2.38) at follow-up. Paired t-tests revealed a significant reduction in maximum driving distance (t = 3.854, p = 0.001) and a significant decline in MoCA scores (t = 2.435, p = 0.024). Pearson correlations showed no significant association between MoCA scores and maximum distance from home at baseline (r = -0.16, p = 0.49) or follow-up (r = -0.12, p = 0.6). The study found that both driving distance and cognitive performance declined over time. Overall, real-world driving behavior offers a longitudinal index to complement clinical assessments in patients at risk for cognitive decline and neurodegeneration.

